# The complete chloroplast genome of *Castanea crenata* Sieb. & Zucc

**DOI:** 10.1080/23802359.2019.1687038

**Published:** 2019-11-08

**Authors:** Min-Jeong Kang, Tae-Dong Kim, Sang-A Lee, Hyo-Ryeon Lee, Chulwoo Kim, Hyoshin Lee, Eung-Jun Park

**Affiliations:** Department of Forest Bio Resources, National Institute of Forest Science, Suwon, Republic of Korea

**Keywords:** *Castanea crenata* Sieb. & Zucc., chloroplast genome, phylogenetic analysis

## Abstract

The complete chloroplast genome sequence of *Castanea crenata* was sequenced and assembled using PacBio Sequel data. The cpDNA was 160,787 bp in length, containing a pair of inverted repeats (IRs) of 25,654 bp each separated by a large and small single copy (LSC and SSC) regions of 90,645 bp and 18,836 bp, respectively. The cpDNA contained 102 genes, including 65 protein-coding genes, 8 ribosomal RNA genes and 37 transfer RNA genes. Phylogenetic analysis indicated that *C. crenata* was closest to *C. pumila var. pumila*, which is known as a typical variety of American chinquapin or dwarf chestnut.

The genus *Castanea*, which belongs to the angiosperm family of *Fagaceae*, is widely distributed in the temperate regions of the Northern Hemisphere. Chestnut species in the *Castanea* can be found in Korea and Japan (*C. crenata* Sieb. & Zucc.), China (*C. mollissima* BL. and *C. seguinii* Dode), North America (*C. dentata* (Marsh.) Brokh), and Europe (*C. sativa* Mill.) (Jaynes 1975). In Korea, most of the native chestnuts have been considered as intermediates between *C. crenata* and *C. mollissima* in terms of their morphological characteristics and nut traits (Kim et al. 2004). In this study, we characterized the complete chloroplast genome sequence of *C. crenata* Sieb. & Zucc. for species identification and phylogenetic analysis.

A plant material of *C. crenata* was collected from Ungyo-ri (37°48´ N 128°34´ E, 475 m above sea level) in Kangwon province, eastern Korea. This chestnut tree, a Korean National Monument (No. 498), is an estimated 370-year-old tree with 14 m in height and 6.4 m in stem diameter. The total DNA was extracted from fresh leaves through nuclei lysis using the CTAB method (Inglis et al. 2018) and stored in the Forest Bio Resources DNA bank, the National Institute of Forest Science. The whole-genome PacBio Sequel DNA sequencing was conducted by DNA Link, Inc. (Seoul, South Korea). A total of 5,077,268 raw reads (mean read length 9550 bp) were retrieved. We performed BWA mapping against three alternative chloroplast genome sequences of *Theobroma cacao* (HQ336404), *Prunus persica* (HQ336405), and *C. mollissima* (HQ336406). The resultant 84,322 filtered reads were then used for the chloroplast genome assembly using the CANU assembler. The annotated genome sequence has been deposited into Genbank under the accession number MN402457.

The circular genome is 160,787 bp in size and comprises a pair of inverted repeat (IR) regions of 25,654 bp each, a large single-copy (LSC) region of 90,645 bp, and a small single-copy (SSC) region of 18,836 bp. The chloroplast genome contains 102 genes, including 65 protein-coding genes (60 PCG species), 8 ribosomal RNA genes (4 rRNA species), and 37 transfer RNA genes (27 tRNA species).

Complete chloroplast genome sequence of *C. crenata* was aligned with chloroplast genome sequences of 18 other plant species in the *Fagaceae* family. The evolutionary history was inferred by using the maximum-likelihood method based on JTT matrix-based model (Jones et al. 1992). The tree with the highest log likelihood (−48,737.43) is shown. Initial trees for the heuristic search were obtained automatically by applying neighbor-join and BioNJ algorithms to a matrix of pairwise distances estimated using a JTT model, and then selecting the topology with superior log-likelihood value. The tree is drawn to scale, with branch lengths measured in the number of substitutions per site. This analysis involved 21 amino acid sequences. Evolutionary analyses were conducted in MEGA X (Kumar et al. 2018). As expected, all *Castanea* chloroplast genome sequences clustered together. Within *Castanea* cluster, *C. crenata* was closely related to *C. pumila var. pumila* (American chinquapin) and *C. sequinii* (Figure 1).

**Figure 1.**
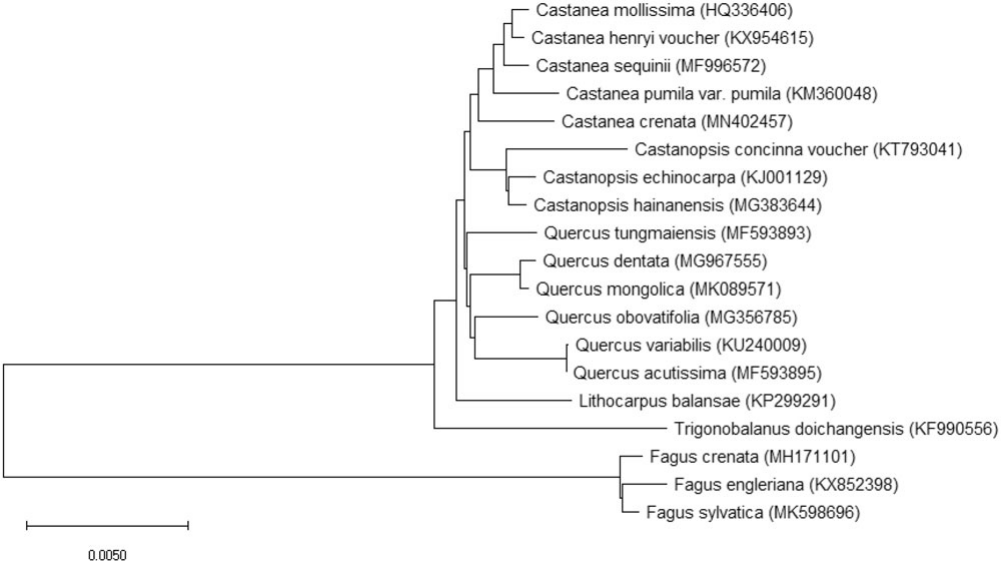
Phylogenetic analysis of *Castanea crenata* Sieb. & Zucc. with 18 members of the Fagaceae family based on the complete chloroplast genome sequences. The evolutionary history was inferred by using the maximum-likelihood method based on the JTT matrix-based model. GenBank accession numbers are shown in parentheses.
